# Knock out of sHSP genes determines some modifications in the probiotic attitude of *Lactiplantibacillus plantarum*

**DOI:** 10.1007/s10529-020-03041-6

**Published:** 2020-11-06

**Authors:** Angela Longo, Pasquale Russo, Vittorio Capozzi, Giuseppe Spano, Daniela Fiocco

**Affiliations:** 1 SAFE Department, University of Foggia, Via Napoli 25, 71122 Foggia, Italy; 2 Institute of Sciences of Food Production, National Research Council (CNR) of Italy, c/o CS-DAT, Via Michele Protano, 71121 Foggia, Italy; 3 Department of Clinical and Experimental Medicine, Universiy of Foggia, Viale Pinto 1, 71122 Foggia, Italy

**Keywords:** Small heat shock protein, Probiotic, Immune-modulation, Microbe-host interaction, Gastro-intestinal stress

## Abstract

**Objective:**

We investigated whether the knock out of small heat shock protein (sHSP) genes (*hsp1*, *hsp2* and *hsp3*) impact on probiotic features of *Lactiplantibacillus plantarum* WCFS1, aiming to find specific microbial effectors involved in microbe-host interplay.

**Results:**

The probiotic properties of *L. plantarum* WCFS1 wild type, *hsp1*, *hsp2* and *hsp3* mutant clones were evaluated and compared through in vitro trials. Oro-gastro-intestinal assays pointed to significantly lower survival for *hsp1* and *hsp2* mutants under stomach-like conditions, and for *hsp3* mutant under intestinal stress. Adhesion to human enterocyte-like cells was similar for all clones, though the *hsp2* mutant exhibited higher adhesiveness. *L. plantarum* cells attenuated the transcriptional induction of pro-inflammatory cytokines on lipopolysaccharide-treated human macrophages, with some exception for the *hsp1* mutant. Intriguingly, this clone also induced a higher IL10/IL12 ratio, which is assumed to indicate the anti-inflammatory potential of probiotics.

**Conclusions:**

sHSP genes deletion determined some differences in gut stress resistance, cellular adhesion and immuno-modulation, also implying effects on in vivo interaction with the host. HSP1 might contribute to immunomodulatory mechanisms, though additional experiments are necessary to test this feature.

**Electronic supplementary material:**

The online version of this article (10.1007/s10529-020-03041-6) contains supplementary material, which is available to authorized users.

## Introduction

Several lactic acid bacteria (LAB) have been claimed probiotics, i.e. “live microorganisms that, when administered in adequate amounts, confer a health benefit on the host” (Hill et al. 2014). *Lactiplantibacillus plantarum* (formerly known as *Lactobacillus plantarum*) belongs to a novel genus which resulted from an updated taxonomical classification of various lactobacilli, a subgroup of LAB (Zheng et al. 2020). *L. plantarum* boasts a vast record of scientific publications and holds tremendous potentialities for biotechnological and biomedical applications. Indeed, this species is widespread in human-associated habitats and participates to various food fermentation processes (Zheng et al. 2020). Moreover, generally recognised as safe, *L. plantarum* comprises strains which are considered beneficial to humans and are included in many commercialised probiotic formulations (Seddik et al. 2017). In particular, *L. plantarum* strain WCFS1 is one of the most extensively studied among probiotic lactobacilli, and it is deemed an invaluable model for studying host-probiotic interactions (Van den Nieuwboer et al. 2016).

The health claims of probiotics need to be substantiated by shedding light on their mode of action and by tracing out the specific microbial effectors involved in the interplay with the host (Lebeer et al. 2018). Among probiotic lactobacilli, some key molecules and structures have been identified which are relevant to their probiotic properties and, not surprisingly, most of them are located on the bacterial cell surface, which is the first site of interaction with the host (Grangette et al. 2005; Murofushi et al. 2015; Tytgat et al. 2016).

The screening and characterisation of probiotics rely on diverse research approaches, including in vitro experiments, genomic profiling along with other omics surveys, in vivo biological models and clinical trials (Papadimitriou et al. 2015). In vitro investigations have major limitations as they do not reflect the complexity of in vivo interactions; however they are straightforward, cost-effective and non-invasive; besides, they permit a strict control of the conditions and the dissection of the single elements involved. Moreover, a fair correspondence between in vivo and in vitro results has been often observed, as for studies on *L. plantarum* (Grangette et al. 2005; Foligne et al. 2007; Štofilová et al. 2017). Straightforward but relevant aspects of probiotics, which can be studied in vitro, include their capacity to survive the gastrointestinal transit, so to reach alive and at efficacious doses the intestine, their ability to persist in the host gut (e.g., by colonising the intestinal mucosa) and their immunomodulatory potential (Papadimitriou et al. 2015).

Small heat shock proteins (sHSP) are ubiquitous, ATP-independent chaperones that act by binding unfolding (substrate) proteins, thereby preventing their irreversible aggregation (Haslbeck et al. 2019). sHSP contribute both to cellular defence against stress and to protein homeostasis under physiological conditions (Haslbeck and Vierling 2015). In a few bacteria, including a LAB species, sHSP have been shown to interact with lipid membranes (Maitre et al. 2012), hence supporting a membrane-stabilising function.

In earlier studies, we have characterised the stress tolerance and membrane properties of the *L. plantarum* WCFS1 clones resulting from the knock out (KO) of its three sHSP genes (Capozzi et al. 2011; Arena et al. 2019). The phenotypic analyses of the mutant clones revealed that sHSP deletion could affect cell surface features and plasma membrane fluidity. Because the cell surface of probiotic microorganisms plays a crucial role in interaction with the animal host, here we sought to assess whether these mutations might have consequences on some relevant probiotic properties of *L. plantarum*, including its gut colonisation ability and immunomodulatory properties. To this aim, we assayed and compared in vitro wild type and mutant clones of *L. plantarum* WCFS1 for survival to an oro-gastro-intestinal (OGI) mimicking model, adhesion to human enterocyte-like cells and capacity to stimulate cytokine gene transcription in human macrophages.

## Methods

### Bacterial strains and growth conditions

Wild type *L. plantarum* WCFS1 (Kleerebezem et al. 2003) and its derivative mutants for *hsp1*, *hsp2*, and *hsp3* (i.e., clones ko1, ko2 and ko3, respectively) (Capozzi et al. 2011; Arena et al. 2019) were used in this work. *L. plantarum* cultures were propagated in de Man Rogosa Sharpe (MRS, Oxoid, UK) broth (pH 6.2), at 30 °C. When required, MRS medium was supplemented with chloramphenicol 10 µg mL^−1^. For solid media, agar was added (15 g L^−1^).

### In vitro oro-gastro-intestinal (OGI) transit assay

Mid-exponential phase cultures of *L. plantarum* (OD_600nm_ 0.8) were harvested by centrifugation and resuspended into sterile saline solution (NaCl 8.5 g L^−1^) at a concentration of 5 × 10^8^ colony forming units (CFU) per mL. The bacterial suspensions were subjected to a system that simulates the oro-gastrointestinal (OGI) transit following a protocol adapted from Bove et al. (2012), as schematically described in Supplementary Figure S1. Briefly, oral stress (step t1) was simulated by adding a lysozyme-containing gastric electrolyte solution (6.2 g L^−1^ NaCl; 2.2 g L^−1^ KCl; 0.22 g L^−1^ CaCl_2_; 1.2 g L^−1^ NaHCO_3_). Then, pepsin (Sigma-Aldrich, St Louis, MO, USA) was added and the pH was progressively reduced to simulate a stomach-like environment (steps t2 and t3). Subsequently, the intestinal environment was mimicked by pH neutralisation and by adding bile salts and pancreatin (Sigma-Aldrich) (step t4). Finally, samples were diluted with an intestinal electrolyte solution (5 g L^−1^ NaCl; 0.6 g L^−1^ KCl; 0.25 g L^−1^ CaCl_2_) to mimic the large intestine (step t5). Samples from the different steps (t0 → t5) of the OGI system were serially diluted and plated on MRS agar to determine viable and cultivable cell counts as CFU. Survival to stress was determined relative to control unstressed samples and calculated as log_10_ (CFU_t0_/CFU_tn_) (CFU_t0_, initial cell count; CFU_tn_, cell count at a specific step of the OGI transit).

### Adhesion assay on Caco-2 cells

Caco-2 cells were grown in DMEM (Sigma-Aldrich) supplemented with 10% (v/v) heat-inactivated fetal bovine serum (FBS), 2 mM l-glutamine (Sigma Aldrich), 100 U mL^−1^ penicillin and 0.1 mg mL^−1^ streptomycin, at 37 °C, in 5% CO_2_. Cells were seeded at a concentration of 10^5^ cells mL^−1^ into 96-well tissue culture-treated plates (Sigma-Aldrich) and there cultivated for 14 days, as previously described (Gheziel et al. 2019), in order to develop steady monolayers. Adhesion tests were performed according to Bove et al. (2012). Briefly, *L. plantarum* cells from mid-exponential phase cultures (OD_600nm_ 0.8) were harvested, washed with phosphate-buffered saline (PBS, pH 7.4), resuspended in absolute DMEM and incubated onto Caco-2 monolayers for 1 h, at 37 °C, with 5% CO_2_ (ratio 1000:1, bacteria to Caco-2 cells). The adhesion percentage was determined by CFU counting, after plating appropriate dilutions of the bacterial suspensions from control and test wells. Three different experiments, run in triplicate, were performed.

### Stimulation of human macrophages and transcriptional analysis

Human monocytoid leukemia-derived cells (THP-1) were purchased from Sigma-Aldrich and propagated in RPMI-1640 (Gibco, Carlsbad, CA) containing 10% (v/v) fetal bovine serum (FBS), 2 mM l-glutamine, 50 U mL^−1^ penicillin and 50 μg mL^−1^ streptomycin, in 5% CO_2_ at 37 °C. For immunostimulation experiments, THP-1 cells were seeded (5 × 10^5^ cells/well) in 24-wells tissue culture-treated plates (EuroClone, Milan, Italy), using unsupplemented medium, and phenotypic differentiation into macrophages was induced by adding 100 ng mL^−1^ phorbol 12-myristate 13-acetate (PMA) (Sigma-Aldrich). After 48 h, plastic adherent THP-1-derived macrophages were exposed to 100 ng mL^−1^ of lipopolysaccharides (LPS) from *Escherichia coli* O127:B8 (Sigma-Aldrich) and co-incubated with live bacterial cells from mid-exponential phase cultures (OD_600nm_ 0.8) in a ratio of 1:1000 (macrophages: bacteria), as previously reported (Arena et al. 2016). Negative and positive controls were unstimulated macrophages and macrophages stimulated only with LPS, respectively. After 3 h incubation, total RNA was isolated from human cells using TRIzol reagent following manufacturer instructions (Ambion, Thermo Fisher Scientific, Waltham, MA), checked for integrity by gel electrophoresis, spectrophotometrically quantified (BioTek Instruments, Winooski, VT) and reverse-transcribed using QuantiTect Reverse Transcription Kit (Qiagen, Valencia, CA). The relative expression level of immune-related genes, i.e., coding for interleukins IL-8, IL-10, IL-12 alpha (IL-12α) and tumor necrosis factor alpha (TNF-α) was performed by quantitative RT-PCR (qRT-PCR) in a real-time instrument (ABI 7300, Applied Biosystems, Foster City, CA), as previously described (Bove et al. 2012). The transcriptional level of β-actin was used to normalise the expression of target genes using the 2^−ΔΔCt^ method. The primers used are listed in supplementary table S1.

### Statistics

The distribution of data was analysed by using the Kolmogorov–Smirnov test of normality. One-way ANOVA followed by post hoc Tukey HSD test was used to analyse data and determine any statistically significant difference (available software at https://astatsa.com), with *p* < 0.05 as the minimal level of significance.

## Results

### Resistance to OGI stress

**Fig. 1:**
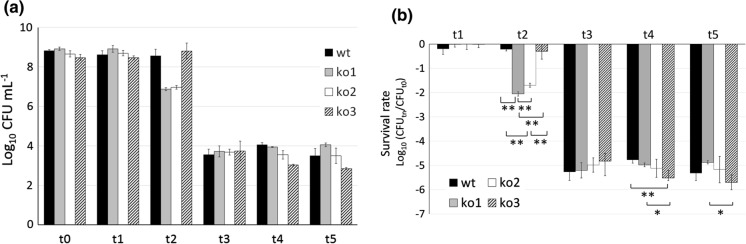
Survival of *L. plantarum* WCFS1 during an in vitro simulated oro-gastro-intestinal (OGI) assay. **a** CFU counts and **b** relative survival of *L. plantarum* wild type (wt) and *hsp* mutant clones (ko1, ko2, ko3) at different steps of the in vitro simulated OGI transit. Data shown are means ± standard deviations. Statistically significant differences between relative survivals at each time point were determined by one-way ANOVA (*p* value set at 0.05) followed by Tukey’s multiple comparison test: **p ≤ 0.01; *p ≤ 0.05

*L. plantarum*, wild type and sHSP mutant clones were challenged in an OGI tract model that mimics, in vitro, the typical stress conditions encountered in the mouth (i.e., presence of lysozyme), in the stomach (i.e., low pH and gastric enzymes), and in the intestine (i.e., neutral pH with pancreatic enzymes and bile salts). CFU count and evaluation of survival at the different steps of the OGI assay (Fig. 1) revealed significant differences between clones at step t2, which corresponds to the first exposure to gastric conditions with highly acidic pH. Indeed, at this stage, *hsp1* and *hsp2* mutants exhibited much lower survival compared to wild type and *hsp3* mutant (i.e. for the former CFU counts decreased by 2 log units, while the latter declined by less than 1 log). Nonetheless, at step t3, when exposure to even lower pH persisted for a longer period, the survival capacity was extremely challenged and become similar for all clones, with a generalised decrease of CFU, by approximately 5–6 log units. Under intestinal conditions, survival seemed to be no more heavily tested. Indeed the viable counts stayed in the range of 10^4^–10^3^ CFU mL^−1^ for all the clones. At these stages, i.e., t4 and t5, it was the *hsp3* mutant to show the least resistance, with significantly lower survival compared to wild type and ko1 clones.

### Adhesion to Caco-2 cells

**Fig. 2:**
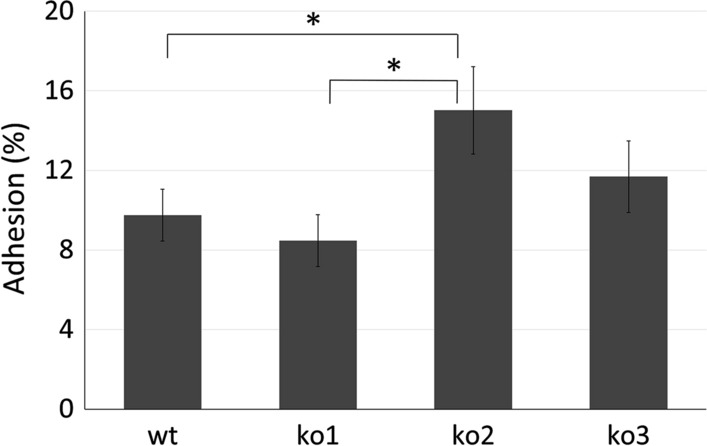
Adhesion of *L. plantarum* WCFS1 cells to Caco-2 monolayers. The adhesion ability was expressed as the percentage of adhesion for cells from wild type (wt) and its derivative mutants for *hsp1* (ko1), *hsp2* (ko2) and *hsp3* (ko3). Values are mean ± SE of three different experiments. Statistically significant differences were determined by one-way ANOVA (*p* value set at 0.05) and Tukey’s multiple comparison test: *p ≤ 0.05

The ability of *L. plantarum* to adhere to cultured human enterocyte-like cells was assayed and the results are shown in Fig. 2. The adhesion assay included the wild type clone and its isogenic mutant for *hsp1*, *hsp2*, and *hsp3*. The adhesion properties of the *hsp2* mutant have been described previously (Bove et al. 2012); however, they have been re-investigated and thus included in this work to allow an easier and direct comparison among the three *hsp* mutants and their original wild type clone. The percentage of adhesion ranged from 8.5 (ko1) to 15.0 (ko2), with some significant differences, as assessed by one-way ANOVA. In detail, ko2 mutant cells exhibited a higher adhesiveness relative to wild type and ko1 clones. The ko3 mutant strain, with an adhesion score of 11.5%, showed an intermediate degree of interaction with cultured human enterocyte-like cells, displaying no significant differences relative to the other three investigated strains.

### Gene expression in LPS-stimulated macrophages

**Fig. 3:**
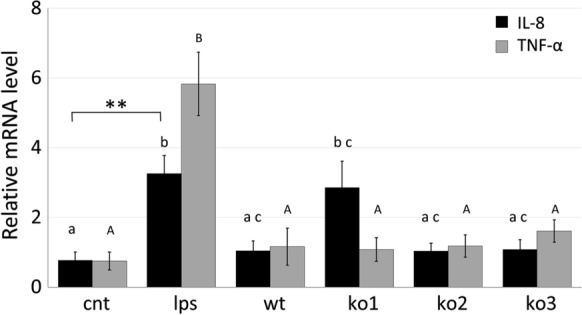
Relative mRNA level of inflammatory cytokines. IL-8 and TNF-α mRNA levels were determined by quantitative real-time RT-PCR in LPS-stimulated macrophages (lps), with or without co-incubation with *L. plantarum* WCFS1 cells from wild type (wt), *hsp1* (ko1), *hsp2* (ko2) and *hsp3* (ko3) mutant clones. Relative mRNA level was obtained by normalising to the transcriptional level observed in unstimulated macrophages (cnt), β-actin gene was used as internal control. Data are mean ± SE from 3 independent experiments. Different superscript letters (lowercase and uppercase for IL-8 and TNF- α, respectively) indicate statistically significant differences between groups as determined by one way ANOVA and post hoc Tukey HSD Test. For difference on IL-8 level *p *< 0.05 and *p *< 0.01, **, as indicated; for difference on TNF-α level, *p *< 0.001

To evaluate and compare the immunomodulatory capacity of *L. plantarum* wild type and *hsp* mutants, LPS-stimulated macrophages were co-incubated with live bacterial cells from the different clones. Then, the transcriptional level of genes encoding cytokines TNF-α, IL-8, IL-10 and IL-12 was analysed by quantitative RT-PCR. *L. plantarum* cells apparently contrasted the pro-inflammatory effect of LPS, as their presence kept the IL-8 and TNF-α mRNA levels similar to that of control, non-LPS stimulated macrophages (Fig. 3). However, treatment with cells from the ko1 clone resulted in a IL-8 mRNA level fairly comparable to that of macrophages stimulated only by LPS. Conversely, TNF-α expression was consistently and similarly attenuated by co-incubation with all the *L. plantarum* clones. Besides, the mRNA level of pro-inflammatory cytokine, both IL-8 and TNF-α, induced by treating with *L. plantarum*, showed no significant differences between the different clones.

**Fig. 4:**
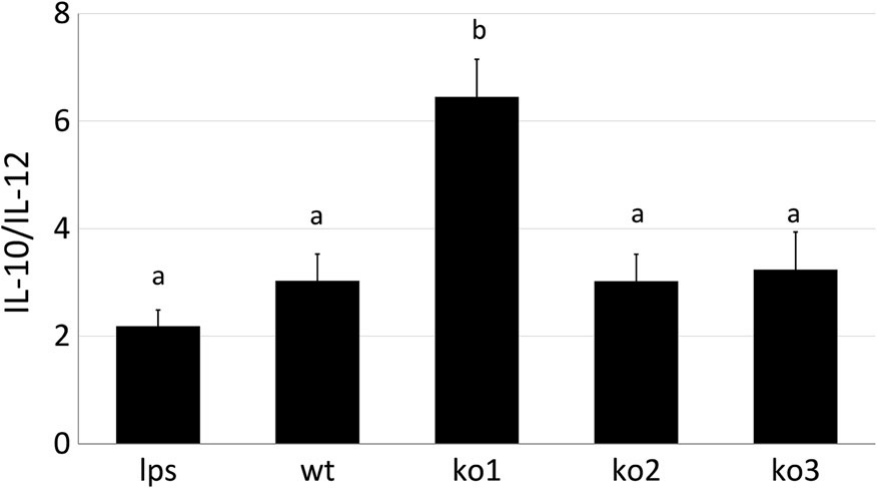
IL-10/IL-12 expression ratio at their transcriptional level. The ratio between IL-10 and IL-12 mRNA levels was determined in LPS-stimulated macrophages. Macrophages were incubated with LPS alone (lps) or with LPS and live cells from *L. plantarum* WCFS1 wild type (wt), or from *hsp1* (ko1), *hsp2* (ko2) and *hsp3* (ko3) mutant clones. The relative mRNA level of the single cytokines was obtained by normalising to the transcriptional level observed in unstimulated macrophages, and β-actin was used as an internal control gene. Data are mean ± SE from 3 independent experiments. Different superscript letters indicate statistically significant differences between groups as determined by one way ANOVA and post hoc Tukey HSD Test (*p *< 0.05)

The *L. plantarum* clones were investigated for their capacities to stimulate macrophages to produce the cytokines IL-10 and IL-12. Because the ratio between the level of anti-inflammatory IL-10 and pro-inflammatory cytokine IL-12 was previously proposed as an indicator to predict the anti-inflammatory properties of lactobacilli (Foligne et al. 2007; Grangette et al. 2005; Van Hemert et al. 2010) the transcriptional profile of these cytokines was investigated in LPS-stimulated macrophages combined or not with *L. plantarum* cells (Fig. 4). By comparing the resulting transcriptional levels, it appears that treatment with ko1 cells resulted in a significantly higher IL-10/IL-12 ratio compared to the other clones, thus suggesting a superior anti-inflammatory potential for this specific mutant.

## Discussion

Insight into the mechanisms underlying probiotic action is still fragmentary. One major issue is the identification of the bacterial effectors specifically involved in the molecular interaction with the host and connected to the health-promoting traits. Extracellular microbial molecules, including cell surface-attached components and released solutes, are presumably crucial for establishing (initial) interactions between probiotics and host cells (Lebeer et al. 2018). In *L. plantarum* WCFS1, the deletion of genes coding for small heat shock proteins (sHSP) affected some cell surface characteristics (Capozzi et al. 2011; Arena et al. 2019), hinting to possible impact on its adhesion and immunomodulatory properties. Moreover, the lack of stress response effectors, such as sHSP, might weaken its resistance to the peculiar stress conditions characterizing the gastro-intestinal tract of the host. Therefore, the aim of this work was to assess, in vitro, whether the absence of each of the three sHSP might have implications on some probiotic attributes of *L. plantarum*, in an attempt to associate distinct microbial factors to specific effects on probiotic traits.

The ability to survive the sequential stresses encountered during passage through the human digestive tract is a relevant feature of probiotic LAB (van Bokhorst-van de Veen et al. 2012), and OGI transit assays are helpful to predict the robustness of probiotics, in view of their use as components of functional foods (Grujović et al. 2019; Fiocco et al. 2020). The extreme acidity encountered in the stomach usually constitutes a major pitfall for orally-ingested, food-borne bacteria, and this was previously demonstrated also for *L. plantarum*, and other probiotic LAB, by using different OGI systems (Bove et al. 2012; van Bokhorst-van de Veen et al. 2012; Arena et al. 2014; Arena et al. 2016). Such observation was corroborated even by the present study, as we found the highest drop in survival, i.e. 5 to 6 log decrease in cell cultivability, just upon exposure to pH 2.0 and gastric enzymes. Indeed, comparable viability levels were observed for other probiotics, when exposed to stomach-like conditions (de Palencia et al. 2008; Arena et al. 2016). The higher sensitivity to gastric stress, as observed in mutants for *hsp1* and *hsp2* (step t2), indicates that these two sHSP may be involved in coping with this kind of stress and partially corroborates previous analyses (Arena et al. 2019), including a significant transcriptional activation of sHSP genes in response to human gastric-like environment (Bove et al. 2013). Intriguingly, after a prolonged exposure to stomach-like conditions, and upon further acidification (i.e., step t3), the survival rate become similar between wild type and mutants. Therefore, it is possible that harshest conditions do not allow to distinguish subtle differences in stress tolerance as those elicited by a milder gastric stress. Alternatively, compensation mechanisms (e.g., by gene reprogramming) may be induced in the mutants, allowing to counteract the adverse conditions with an efficacy that is similar to that of the wild type. Under intestine-resembling conditions, the ko3 mutant exhibited lower survivals, suggesting that HSP3 might be specifically required for managing bile stress, which is in line with previous data (Bove et al. 2013; Arena et al. 2019).

Adherence to intestinal epithelial cells is desirable for probiotics, as it facilitates colonisation of the host gut, thereby enhancing intestinal barrier function and antagonism against pathogens. Human enterocyte-like Caco-2 cells are usually employed for in vitro adhesion assays (Messaoudi et al. 2012; Bove et al. 2012), which were shown to provide a good prediction of in vivo results (Crociani et al. 1995). The levels of adhesion observed in the present work are consistent with previous studies on *L. plantarum* WCFS1 (Bove et al. 2012; Arena et al. 2014; Lee et al. 2016; Gheziel et al. 2019) and on correlated lactobacilli species (Xu et al. 2009; Messaoudi et al. 2012; Arena et al. 2014). Knock out of *hsp1* and, to a minor extent, of *hsp3* was found to reduce cell surface hydrophobicity and decrease biofilm formation on abiotic substrates (Arena et al. 2019). Yet, present data indicate that the lack of *hsp1* or *hsp3* has no relevant consequence on the adhesion properties of *L. plantarum* cells on a biotic surface (such as that constituted by enterocyte-like monolayers), suggesting neglectable effects on host gut colonisation ability, in vivo. Indeed, based on previous studies, we speculate that *hsp1* and *hsp3* mutants of *L. plantarum* WCFS1 might possess higher or similar in vitro adhesiveness compared to some commercial probiotics, such as *Lactobacillus acidophilus* LA5 and *Bifidobacterium lactis* Bb-12 (de Palencia et al. 2008; Arena et al. 2016). On the other hand, the present work confirms an increased adhesion to cells for the *hsp2* mutant (Bove et al. 2012), which also exhibited some modifications of its cell surface properties (Capozzi et al. 2011). Then, our findings also confirm that cell envelope hydrophobicity and biofilm formation do not always reflect the adhesive strength on animal cells (Papadimitriou et al. 2015). Indeed, sometimes, cell surface hydrophobicity was found to well-correlate to the adhesive phenotype of lactobacilli (Xu et al. 2009; Grujović et al. 2019). However, this latter capacity is also influenced by strain and conditions; therefore, data on cell surface physicochemical properties may not be reliable enough to predict adhesiveness on biotic surfaces (Savage 1992).

The health benefits ascribed to probiotics usually pertain their capacity to modulate host immunity. This feature can be studied in vitro by evaluating the level of cytokines and/or secretory immunoglobulins produced by human immune cells, following bacterial stimulation. Because the pattern of cytokine induced by probiotics is variable and mostly strain-specific (Foligne et al. 2007; Meijerink et al. 2010), this allows to identify microbial strains with immunomodulatory properties and, among them, those endowed with either pro- or anti-inflammatory effects (Meijerink et al. 2010; Garcia-Gonzalez et al. 2018; Gheziel et al. 2019). In this regard, the IL-10/IL12 ratio has been proven useful to preliminarily estimate the anti-inflammatory potential of probiotic lactobacilli (Grangette et al. 2005; Foligne et al. 2007; Van Hemert et al. 2010).

In human macrophages stimulated with only LPS, as expected, a pronounced induction of typical pro-inflammatory markers, such as TNF-α and IL-8, was detected. When macrophages were stimulated with LPS combined with live *L. plantarum* cells, the transcriptional induction of these pro-inflammatory signals was consistently attenuated. This would happen in presence of cells from all the *L. plantarum* clones, except for the ko1 mutant in relation to IL-8 mRNA. Such expression pattern points to the anti-inflammatory action of *L. plantarum* cells in vitro and to its immune regulative potentials in vivo, being in line with previous studies (Foligne et al. 2007; Bäuerl et al. 2013; Arena et al. 2016; Gheziel et al. 2019). Earlier analyses focusing on citokyne stimulation showed that the anti-inflammatory potential of *L. plantarum* is comparable to, and sometimes greater than, that of other probiotic species (Bäuerl et al. 2013; Arena et al. 2016), despite being considerably variable among different *L. plantarum* strains (Meijerink et al. 2010; van Hemert et al. 2010; Garcia-Gonzalez et al. 2018). Then, our data indicate that the deletion of *hsp2* and *hsp3* does not modify the capability of *L. plantarum* WCFS1 to counteract in vitro a pro-inflammatory stimulus (i.e., by LPS). Therefore, the cell surface modifications possibly associated to such mutations do not impact on structures and molecules that may be sensed by host immune cells, e.g. through their pattern recognition receptors (Lee et al. 2006). Intriguingly, the ko1 clone was less effective in attenuating the pro-inflammatory LPS-deriving stimulus, as observed in relation to IL-8 mRNA; whereas TNF-α mRNA repression was consistent also upon treatment with cells from this clone. Moreover, when we evaluated the IL-10/IL-12 ratio, incubation with ko1 cells resulted in a significantly higher value, thus suggesting that the ko1 clone could actually hold a greater anti-inflammatory potential. The IL-10/IL12 ratio induced by *L. plantarum*, relative to secreted cytokines, was previously estimated low in comparison to other probiotic strains (Foligne et al. 2007). This finding, then, hints to the possibility that lack of HSP1 could determine some subtle changes in the interaction between microbial and host cells, e.g. by increasing the exposure of some surface components and/or the release of soluble compounds that elicit an anti-inflammatory reaction. Alternatively, HSP1 loss might promote the shielding of some cell envelope-associated molecules with a pro-inflammatory character. This could relate to a general protein homeostasis activity ascribed to sHSP (Capozzi et al. 2011; Haslbeck and Vierling 2015), as well as reflect the membrane-fluidising effect hypothesised for HSP1 in *L. plantarum* WCFS1 (Arena et al. 2019). Besides, a direct involvement of HSP1 in signalling mechanisms cannot be ruled out. For instance, another chaperone, i.e., the HSP GROE, was identified as an immunomodulatory protein of *Lactobacillus casei* (Rieu et al. 2014). However, further experiments would be necessary to prove this, including isolation and purification of *L. plantarum* HSP1. Next, it will be worth studying the probiotic properties of *L. plantarum* double KO mutants for the sHSP genes, in order to assess, for instance, the combined effect of the lack of both HSP1 and HSP2.

## Conclusion

Earlier studies have sought to identify the genetic loci of *L. plantarum* that may be relevant for its interaction with the host (Meijerink et al. 2010; Van Hemert et al. 2010; Bove et al. 2012; Lee et al. 2016). In some cases, the mutation of single genes was found to significantly affect some aspects of its probiotic activity; consequently, distinct genes could be associated to specific health-promoting features, especially concerning the immunomodulatory capacity (Grangette et al. 2005; Meijerink et al. 2010; Lee et al. 2016). To our knowledge, this is the first study that evaluates how the single deletion of a family of sHSP genes affects some selected probiosis-related characteristics. Here, the genetic background of the examined *L. plantarum* WCFS1 *hsp* mutants slightly altered its capacity to resist to OGI stress, adhesion to enterocytes and immuno-modulation of macrophages. The lack of prominent effects might depend on a compensation of the genetic loss and/or indicate that neither of the three sHSP has a direct, essential function in the investigated properties. Yet, the alterations observed in vitro might affect also the interaction with the host, in vivo. Interestingly, the effects of *hsp* mutation were diversified, thus suggesting that the single sHSP might contribute to different aspects of the probiotic phenotype. Noticeably, a possible role for HSP1 emerged in the stimulation of host immune cells and this aspect shall deserve further investigation.

## Electronic supplementary material

Below is the link to the electronic supplementary material.

Supplementary material 1 (DOCX 78 kb)
