# Supplementary material for: Knock out of sHSP genes determines some modifications in the probiotic attitude of Lactiplantibacillus plantarum
Source: Biotechnol Lett. 2020 Nov 6;43(3):645–54. doi: 10.1007/s10529-020-03041-6 (PMC7872990; doi:10.1007/s10529-020-03041-6)
Supplement: Supplementary file 1 — Supplementary material 1 (DOCX 78 kb) [file 10529_2020_3041_MOESM1_ESM.docx]

Supplementary material


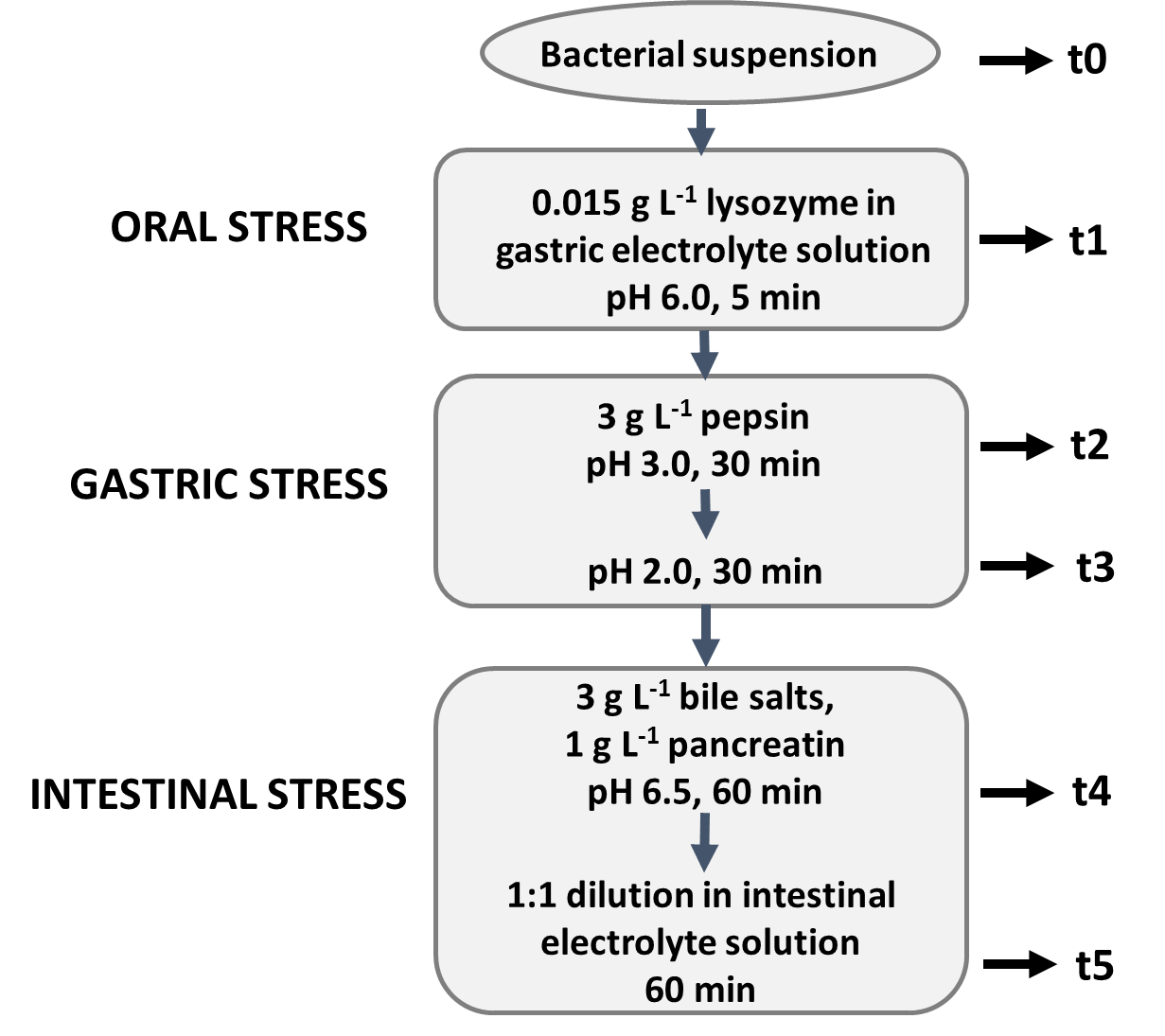


**Figure S1.** Scheme of the *in vitro* system simulating the transit along the human oro-gastro-intestinal tract. Bacterial cells were subjected to the sequential conditions indicated. Incubations were performed for the time indicated, at 37 °C and under shaking. Unstressed sample (t0) was considered as internal control.

**Table S1**. Oligonucleotides used in this study

| **Primer name** | **Sequence (5’-3’)** | **Target gene, sequence ID** |
| --- | --- | --- |
| β-act F | AAAGACCTGTACGCCAACAC | β-actin, [NM_001101.4](https://www.ncbi.nlm.nih.gov/nucleotide/NM_001101.4?report=genbank&log$=nucltop&blast_rank=1&RID=SPG3CKBB01R) |
| β-act R | CATACTCCTGCTTGCTGATCC |  |
| IL-8 F | TGTGGAGAAGTTTTTGAAGAGGG | interleukin 8, NM_000584.3 |
| IL-8 R | CCAGGAATCTTGTATTGCATCTGG |  |
| IL-10 F | GACTTTAAGGGTTACCTGGGTTG | interleukin 10, NM_000572.2 |
| IL-10 R | TCACATGCGCCTTGATGTCTG |  |
| IL-12α F | GATGGCCCTGTGCCTTAGTA | Interleukin 12α (p35), NM_001354582.1 |
| IL-12α R | TCAAGGGAGGATTTTTGTGG |  |
| TNF-α F | AACCTCCTCTCTGCCATCAA | tumor necrosis factor-α, NM_000594.3 |
| TNF-α R | ATGTTCGTCCTCCTCACAGG |  |
